# Interrogation of *IDH1* Status in Gliomas by Fourier Transform Infrared Spectroscopy

**DOI:** 10.3390/cancers12123682

**Published:** 2020-12-08

**Authors:** James M. Cameron, Justin J. A. Conn, Christopher Rinaldi, Alexandra Sala, Paul M. Brennan, Michael D. Jenkinson, Helen Caldwell, Gianfelice Cinque, Khaja Syed, Holly J. Butler, Mark G. Hegarty, David S. Palmer, Matthew J. Baker

**Affiliations:** 1 WestCHEM, Department of Pure and Applied Chemistry, Technology and Innovation Centre, University of Strathclyde, 99 George St., Glasgow G1 1RD, UK; james.cameron@clinspecdx.com (J.M.C.); christopher.rinaldi@strath.ac.uk (C.R.); alexandra.sala@clinspecdx.com (A.S.); 2 ClinSpec Diagnostics, Technology and Innovation Centre, University of Strathclyde, 99 George St., Glasgow G1 1RD, UK; justin.conn@clinspecdx.com (J.J.A.C.); holly.butler@clinspecdx.com (H.J.B.); mark.hegarty@clinspecdx.com (M.G.H.); david.palmer@strath.ac.uk (D.S.P.); 3 Department of Clinical Neurosciences, Translational Neurosurgery, Western General Hospital, Edinburgh EH4 2XU, UK; paul.brennan@ed.ac.uk; 4 Institute of Systems, Molecular and Integrated Biology, University of Liverpool & The Walton Centre NHS Foundation Trust, Lower Lane, Fazakerley, Liverpool L9 7LJ, UK; Michael.Jenkinson@liverpool.ac.uk; 5 Institute of Genetics and Molecular Medicine, University of Edinburgh, Division of Pathology, Western General Hospital, Crewe Road South, Edinburgh EH4 2XR, UK; h.caldwell@ed.ac.uk; 6 Diamond Light Source, Harwell Science and Innovation Campus, Chilton, Oxfordshire OX11 0DE, UK; gianfelice.cinque@diamond.ac.uk; 7 Walton Research Tissue Bank, Neurosciences Laboratories, The Walton Centre NHS Foundation Trust, Lower Lane, Fazakerley, Liverpool L9 7LJ, UK; khaja.syed@thewaltoncentre.nhs.uk; 8 WestCHEM, Department of Pure and Applied Chemistry, Thomas Graham Building, University of Strathclyde, 295 Cathedral Str., Glasgow G1 1XL, UK

**Keywords:** biophotonics, infrared, imaging, cancer, histopathology, biofluids, glioma

## Abstract

**Simple Summary:**

Gliomas represent the vast majority of primary brain tumours and are of significant medical importance due to the poor clinical course of affected patients. The isocitrate dehydrogenase 1 (*IDH1)* mutation is associated with improved prognosis, compared to patients with *IDH1*-wildtype lesions of the same stage. In this proof-of-concept study, Fourier transform infrared spectroscopy was used to determine the *IDH1* molecular status in fixed glioma sections. Classification algorithms successfully distinguished the two *IDH1* classes with high accuracies (>80%). Knowledge of the *IDH1* status would be beneficial, as maximum resection may be preferred in patients with *IDH1*-mutant gliomas, whilst a more limited resection can be best for *IDH1*-wildtype gliomas. Furthermore, we examined blood serum in an attempt to identify the biomolecular alterations caused by the *IDH1* mutation. Non-invasive approaches that can detect the molecular status may guide some patients to an alternative treatment prior to surgery.

**Abstract:**

Mutations in the isocitrate dehydrogenase 1 (*IDH1*) gene are found in a high proportion of diffuse gliomas. The presence of the *IDH1* mutation is a valuable diagnostic, prognostic and predictive biomarker for the management of patients with glial tumours. Techniques involving vibrational spectroscopy, e.g., Fourier transform infrared (FTIR) spectroscopy, have previously demonstrated analytical capabilities for cancer detection, and have the potential to contribute to diagnostics. The implementation of FTIR microspectroscopy during surgical biopsy could present a fast, label-free method for molecular genetic classification. For example, the rapid determination of *IDH1* status in a patient with a glioma diagnosis could inform intra-operative decision-making between alternative surgical strategies. In this study, we utilized synchrotron-based FTIR microanalysis to probe tissue microarray sections from 79 glioma patients, and distinguished the positive class (*IDH1*-mutated) from the *IDH1*-wildtype glioma, with a sensitivity and specificity of 82.4% and 83.4%, respectively. We also examined the ability of attenuated total reflection (ATR)-FTIR spectroscopy in detecting the biomolecular events and global epigenetic and metabolic changes associated with mutations in the *IDH1* enzyme, in blood serum samples collected from an additional 72 brain tumour patients. Centrifugal filtration enhanced the diagnostic ability of the classification models, with balanced accuracies up to ~69%. Identification of the molecular status from blood serum prior to biopsy could further direct some patients to alternative treatment strategies.

## 1. Introduction

Somatic mutations in the human cytosolic isocitrate dehydrogenase 1 (*IDH1*) gene are a frequent feature observed in gliomas. The *IDH1* mutation tends to occur in the early stages of gliomagenesis, hence it is most commonly found in low-grade gliomas, diffuse astrocytoma and oligodendrogliomas [1], but is less common (10%) in primary glioblastoma (GBM) [2,3], except where the GBM develops from a previously diagnosed diffuse or anaplastic astrocytoma (>80%) [4,5]. Consequently, the *IDH1* mutation serves as a valuable diagnostic marker (Table 1) by assisting in the differentiation of tumour entities that are often indistinguishable through histopathological analysis alone, but have different treatments and prognostic profiles [5].

The normal function of the *IDH1* enzyme is to convert isocitrate to α-ketoglutarate (αKG). Cancer-associated mutations in *IDH1* inactivate this standard enzymatic activity, but enable a neomorphic conversion of αKG to the oncometabolite 2-hydroxyglutarate (2HG) [7,8]. This results in an accumulation of 2HG in the glioma cells, which is thought to drive oncogenic activity and tumorigenesis [9]. The vast majority (~90%) of *IDH1* mutations involve transitions in codon 132, where the arginine residue is replaced by histidine (R132H-*IDH1*) [2]. Patients who have this *IDH1* mutation in their glioma have a significantly better prognosis compared to those with *IDH1*-wildtype lesions of the same histologic grade [10,11]. For example, those with an *IDH*-mutant GBM tend to have a better predicted prognosis than patients with a lower-grade *IDH*-wildtype astrocytoma [4,12].

The presence of R132H-*IDH1* can be established through immunohistochemistry (IHC) by applying the *mIDH1R132H* antibody to resected glioma tissue [13]. IHC depends on invasive biopsy, with inherent risk for the patient. The R132H-*IDH1* expression may be present only in a fraction of tumour cells in some diffuse gliomas, therefore a negative result does not necessarily exclude a glial lesion as the concentration of immuno-positive diffuse astrocytomas ranges between 50% and 70% [14]. Thus, several sections of tumour often need to be biopsied for a reliable result. Likewise, false positives are occasionally observed because of non-specific background staining, and the regional heterogeneity of R132H-*IDH1* expression can cause doubt in the diagnosis, which may necessitate confirmatory genetic analysis [15].

The development of a simple, rapid and label-free diagnostic tool for *IDH1* detection would be transformative for molecular diagnosis. Analytical techniques involving vibrational spectroscopy have great potential for diagnosing disease states, namely infrared and Raman spectroscopy [16,17,18]. In particular, Fourier transform infrared (FTIR) spectroscopy has been shown to be valuable for the detection of various cancers, such as breast, lung, colorectal, ovarian and prostate cancer [19,20,21,22,23,24,25,26], since it can probe the biochemical composition of normal and pathological tissue and generate the fingerprint structure of several biomolecular components, such as proteins, lipids and nucleic material [27,28]. Several studies have looked into diagnosing brain lesions, utilizing Raman spectroscopy [29,30,31,32]. Likewise, FTIR can detect and stratify brain malignancies through the analysis of resected tissue sections, mainly focused on transmission techniques [33,34,35,36]. On the other hand, attenuated total reflection (ATR)-FTIR is well-suited to biological fluids, such as blood serum [37,38]. The technique provides for the qualitative interrogation of all infrared active macromolecular constituents of blood serum, and it is well established that biomolecular imbalances in biofluids can give an indication of disease states [28]. The plethora of spectroscopic studies highlight the capability of FTIR to become a powerful tool in the diagnostic field [39]. Uckermann et al. recently indicated FTIR could be suitable in identifying mutated *IDH1* expression, through the analysis of 34 frozen brain tissue cryosections and 64 fresh unfixed glioma biopsies [40]. Despite some promising results, the spectra of both the cryosections and fresh tissue biopsies demonstrated high inter-patient variability. The variance in the fresh tissue analysis may have been accentuated by the use of ATR-FTIR, which only interrogates the region of the tissue sample that is in contact with the internal reflection element (IRE), so it can be difficult to ensure that the sampling area being examined is representative of the tumour. Uckermann et al. proposed that further work would be required to fully evaluate the ability of the technique in the application of detecting the *IDH1* mutation and other potential biomarkers. Synchrotron radiation-based FTIR (SR-FTIR) microspectroscopy is a method that can be used to extract finer spatial and spectral details from biological tissue samples [28]. In SR-FTIR, a synchrotron source emits a collimated light beam more intense than standard bench-top spectrometers [41,42]. Synchrotron radiation can be up to $10^{3}$ times brighter than any other conventional broadband IR source, allowing smaller regions of tissue to be probed with superior signal-to-noise [43]. Thus, SR-FTIR spectroscopy offers a high-resolution approach that can be valuable for proof-of-principle studies in acquiring greater biological information.

The implementation of FTIR spectroscopy during surgical biopsy could present a fast, label-free method for the molecular genetic classification of gliomas. *IDH1* mutation is associated with a better prognosis, as is maximal surgical resection [44]. Attempted maximum safe surgical resection may be more justified in patients with *IDH1*-mutant gliomas, whilst a more limited resection may be more appropriate for *IDH1*-wildtype gliomas. Intra-operative rapid determination of tumour *IDH1* status could therefore inform neurosurgical decision-making [45]. In this study, SR-FTIR has been used to examine human brain glioma tissue, where single-point spectra have been collected from tissue microarray (TMA) sections comprising *IDH1*-mutated and *IDH1*-wildtype glioma tissue cores. Additionally, we examine the potential for earlier molecular subclassification of tumours by identifying the biomolecular alterations caused by the genetic *IDH1* mutation in glioma patient serum. The combination of centrifugal filtration and ATR-FTIR serum spectroscopy could be implemented prior to biopsy or resection to determine *IDH1* status even before surgery.

## 2. Materials and Methods

### 2.1. Sample Collection

#### 2.1.1. Glioma Tissue

**Figure 1:**
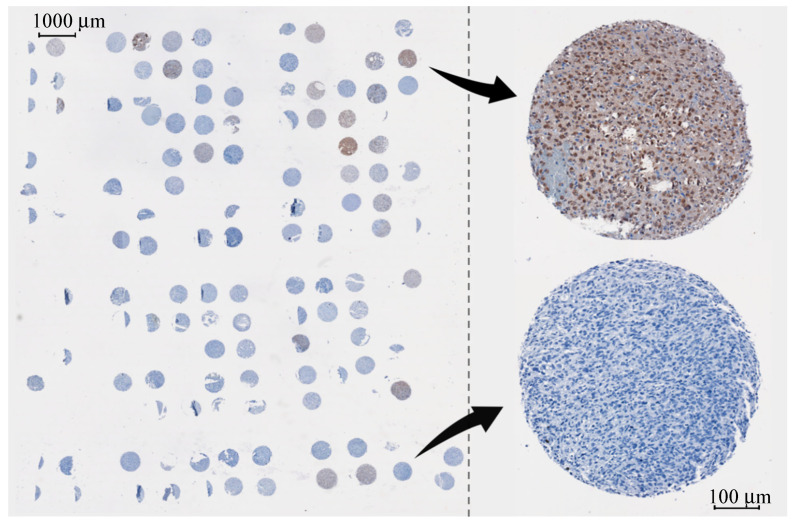
Overview of the tissue microarray with *IDH1* staining, with focus on a mutated core (brown) and wildtype core (blue).

Formalin-fixed paraffin-embedded (FFPE) tumour tissue was obtained from patients who underwent neurosurgery (e.g., tumour biopsy or debulking) with a histologically confirmed glioma as diagnosed by a consultant neuropathologist. In total, 137 patients were selected for inclusion in the TMA from an institutional database of all surgical neuro-oncology patients, in order to represent a range of tumour grades and histological subtypes, as well as matched samples from recurrent tumours. Anonymised clinical information was available. Ethical approval for the construction of the TMA was from the Lothian NRS Bioresource (15 ES 0094). The microarray was constructed from 0.6 mm diameter cores of the FFPE tissue, which were inserted into a recipient block using a manual tissue arrayer. Sections of the TMA block 10 µm thick were sliced with a microtome and floated onto three 76 × 26 × 1 mm calcium fluoride (${\mathrm{CaF}}_{2}$) substrates in a heated water bath (~40 °C). The ${\mathrm{CaF}}_{2}$ slides were placed into an automated Leica ST5010 Autostainer XL (Leica, Wetzlar, Germany) for a dewaxing protocol, designed to remove the paraffin wax before spectroscopic analysis, which proceeded as follows: immersion in xylene (3 × 5 min), ethanol wash (2 × 2 min 100%, 1 × 2 min 80%, 1 × 2 min 50%), rinse in distilled water (2 × 2 min). The dewaxed slides were placed into an oven at 60 °C for 3 h to dry the samples efficiently onto the ${\mathrm{CaF}}_{2}$ substrates. The quantity of each tissue core within the TMA block varied, and thus a small portion of the patients could not be sampled. Once dehydrated, the slides were stored in petri dishes at room temperature until the time of IR interrogation. IHC staining of the tissue cores was separately performed on 4 µm slices using the *mIDH1R132H* antibody. Reference microscope images were collected, allowing the characterisation of the *IDH1*-status of each sample; a positive result is indicated by a strong brown colour in the glioma cells (Figure 1).

#### 2.1.2. Patient Serum

In total, 72 serum samples were obtained from the Walton Centre NHS Trust biobank (Liverpool, UK) from patients with a pathologically confirmed glioma, prior to receiving any chemotherapy or radiotherapy. Ethical approval was obtained (Walton Research Bank and BTNW/WRTB 13_01/BTNW Application #1108). Blood samples had been gathered in serum collection tubes and allowed to clot for up to one hour. The tubes had then been centrifuged for 15 min at 2200× *g*. The serum component was subsequently aliquoted then stored in a −80 °C freezer until the time of analysis.

##### Centrifugal Filtration

To assess whether ATR-FTIR spectroscopy could detect *IDH1* mutation, centrifugal filtration was undertaken to enable analysis of the low molecular weight (LMW) fraction of the serum samples. The whole serum from the 72 brain cancer patients (Table S1) was filtered to remove the more abundant high molecular weight (HMW) biomolecules. Commercially available Amicon Ultra 0.5 mL centrifugal filtering devices (Millipore-Merck, Darmstadt, Germany) with cut-off points at 3 kDa were used to fractionate the serum samples. The serum was split into two fractions: the ‘filtrate’ and the ‘concentrate’. The filtrate accounts for the biomolecular components below the 3 kDa cut-off point, and the concentrate represents the higher molecular weight serum constituents. Serum from each patient (0.3 mL) was placed in the centrifugal filters, and the filtration tubes were centrifuged for 30 min at a speed of 14,000× *g*. The filtrates passed through the membranes into the collection vials. The filters were then inverted and centrifuged for 2 min at 1000× *g* to collect the HMW concentrates. The filtrates and concentrates were stored in a −80 °C freezer until the time of analysis.

### 2.2. Spectral Collection and Data Analysis

#### 2.2.1. Synchrotron Radiation-Based FTIR Microspectroscopy

Experiments were carried out at the Diamond Light Source synchrotron facility, UK, namely on the Multimode Infrared Imaging and Microspectroscopy (MIRIAM) B22 beamline [46,47]. FTIR microspectra were acquired in transmission mode via a Hyperion 3000 microscope system with a 36× magnification (NA = 0.5) Cassegrain objective/condenser optics coupled to a Bruker Vertex 80v FTIR spectrometer (Bruker Optics, Ettlingen, Germany). A high sensitivity liquid nitrogen cooled mercury cadmium telluride (MCT) single element detector with a 50 mm pitch size was used to collect data between 4000 and 600 cm^−1^, at a spectral resolution of 4 cm^−1^. Background spectra were recorded from clean sections on the ${\mathrm{CaF}}_{2}$ substrates. The aperture size was set to have a projected detection area of 10 × 10 μm on the sample plane, with FTIR spectral acquisition performed by co-addition of 256 background scans and 128 sample scans at an FTIR nominal scanner rate of 80 kHz (equivalent to 10 s and 20 s per point, respectively). Point spectra were collected as linescans through diagonal cross-sections of the TMA cores, acquiring approximately ~70 spectra for each TMA core across a line approximately 0.6 mm in length (this ranged from around 40–80 spectra depending on the size and quality of each core). In total, 8532 spectra were accumulated from 99 TMA cores from two ${\mathrm{CaF}}_{2}$ slides of consecutive tissue sections (Table S2), comprised of tumour tissue from 79 glioma patients. Each transmission spectrum was ratioed to the background spectrum and converted to absorbance.

An initial atmospheric compensation was performed to subtract the contribution of spectral water vapour bands in OPUS 8 software (Bruker Optics, Ettlingen, Germany), and the resulting spectra were cut to 4000–900 cm^−1^. The spectral data was exported for further pre-processing and analysis. Absorbance spectra collected from clean sections of the ${\mathrm{CaF}}_{2}$ substrates were subtracted from sample absorbance spectra. The PRFFECT toolbox on the R Statistical Computing Environment was utilised for the pre-processing and classifications [16]. Iterative extended multiplicative signal correction (EMSC) was applied five times with five different reference spectra to account for Mie scattering [48]. Principal component analysis (PCA) was employed on Quasar software (Orange Data Mining [49]) for a PCA-based quality test, to remove spectra that fell outside the central cluster of PC scores. Thereafter, spectra with Amide I (1650 cm^−1^) absorbance <0.01 or >2 were removed from the dataset, as where the absorbance is <0.01 the spectral sensitivity decreases by two orders of magnitude, whereas above >2 the linearity of the detection is compromised, e.g., the stronger signal of typically Amide I cannot be ratioed properly to the other IR bands. The synchrotron datasets were classified based on *IDH1* status using linear discriminant analysis (LDA). LDA is a dimensionality reduction technique which can work as a linear classifier, and it focuses on maximising the separability among the known categories. LDA classifiers make predictions by estimating the probability that a new set of inputs belong to each category, and the class that gets the highest probability is predicted as the output class [50]. Firstly, a grid search was utilised to test various pre-processing parameters, then the top 10 models were further examined with a greater number of iterations. More information on the grid search and the iterations process is contained in the results section. Model performance is reported in terms of sensitivity and specificity. These values are calculated based on the number of correct and incorrect predictions in the external test set; the sensitivity refers to the ability to correctly identify the *IDH1*-mutated samples, whereas the specificity is the ability to successfully pick out the *IDH1*-wildtype patients. We direct the reader to our previous work for more information on the statistical analysis employed here [51].

#### 2.2.2. ATR-FTIR Spectroscopy

The frozen serum samples were thawed at room temperature prior to spectral analysis. Then, 3 µL deposits were pipetted onto each of the three sample wells on a ClinSpec Dx optical sample slide for spectroscopic analysis (ClinSpec Diagnostics Ltd., Glasgow, UK) [52]. The first well remained clean for a background measurement, in order to account for atmospheric conditions [53]. The serum drops were dehydrated in a drying unit incubator (Thermo Fisher Scientific, Massachusetts, USA) at 35 °C for 1 h [53,54,55].

A Perkin Elmer Spectrum 2 FTIR spectrometer (Perkin Elmer, London, UK) coupled with a ClinSpec Dx indexer accessory (ClinSpec Diagnostics Ltd., Glasgow, UK) was used for the spectral collection. The spectra were acquired in the range 4000–450 cm^−1^, at a resolution of 4 cm^−1^, with 1 cm^−1^ data spacing and 16 co-added scans. Each sample well was analysed in triplicate, acquiring 9 spectra per patient. Thus, we gathered 648 whole serum spectra, and 648 spectra were collected from the <3 kDa filtrates, resulting in 1296 spectra in total.

An EMSC was also employed for the serum data analysis. The ‘whole serum’ dataset used a human pooled serum reference, followed by a spectral cut to 1800–1000 cm^−1^. To develop the classification models, patients were randomly split into training and test sets at a 70:30 split. Spectra from a single patient’s serum could only appear in one cross-validation fold, and in either the training or test set. The majority vote amongst the nine spectra for each patient was reported as either *IDH1*-mutated or *IDH1*-wildtype. The classification models were retrained and tested on 100 different randomly selected training and test set partitions to provide a more reliable result. Classification results of the ATR-FTIR spectra from random forest (RF), partial least squares–discriminant analysis (PLS-DA), and support vector machine (SVM) analysis have been compared here, as described in our previous work [51,56].

##### Centrifugal Filtration

For the centrifugal filtration study, the spectra were initially corrected with EMSC using an averaged filtrate spectrum as the reference. Since there were two prominent bands present between 1000 and 800 cm^−1^ in the filtered serum spectrum, the dataset was cut down to 800 cm^−1^ to ensure all potentially important biological information was retained. Thus, three spectral cuts were tested: 4000–800 cm^−1^, 1800–800 cm^−1^ and 1800–1000 cm^−1^. All other parameters were consistent with the whole serum analysis.

## 3. Results

### 3.1. Synchrotron Microanalysis

**Figure 2:**
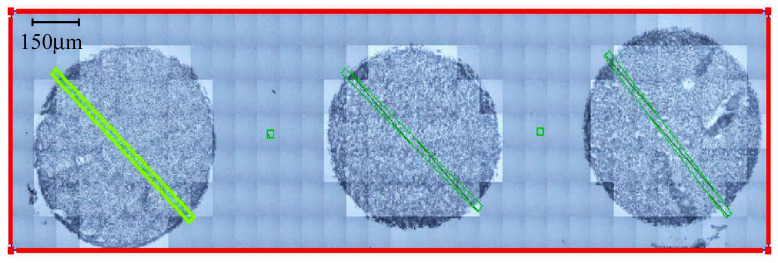
Microscope image taken of three brain tumour tissue microarray cores prior to infrared interrogation. Green squares (10 × 10 μm) represent the points where spectra were collected.

**Figure 3:**
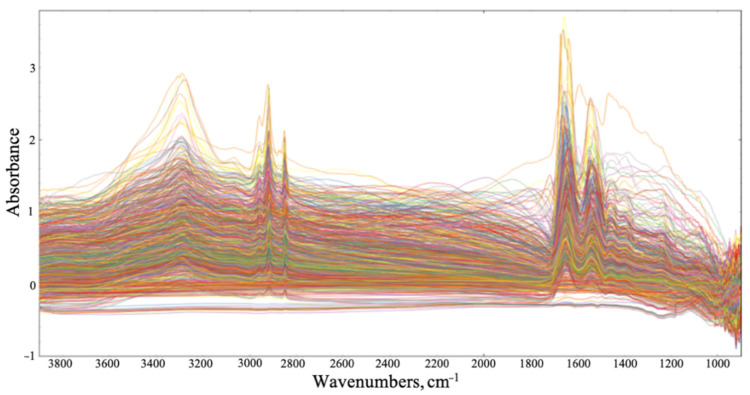
1000 randomly selected raw spectra from the synchrotron dataset displaying highly variable baselines and scattering effects.

**Figure 4:**
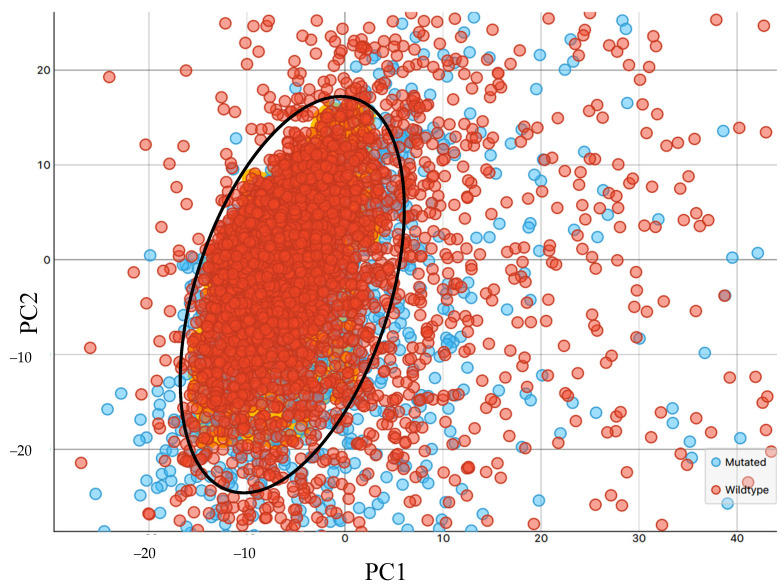
Principal component analysis (PCA)-based quality test: PCA scores plot of principal component 1 (PC1) and principal component 2 (PC2) with focus on centre of the cluster highlighting the ellipse (black circle) containing the data that were carried forward for investigation; all spectral datapoints laying outside of the ellipse were removed from subsequent analysis.

**Figure 5:**
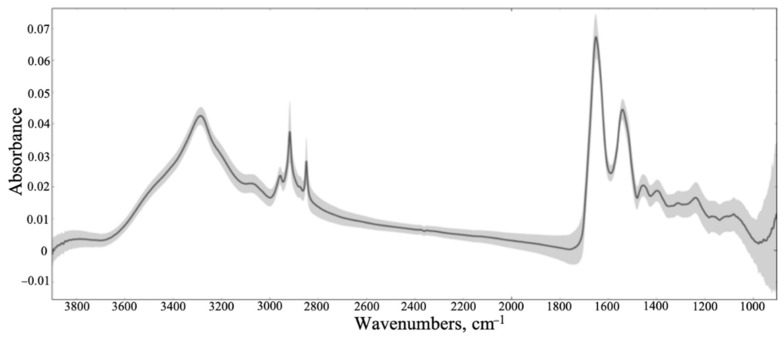
Mean spectra of all samples combined after extended multiplicative signal correction (EMSC), principal component analysis (PCA) quality test and removal of Amide I outliers with the standard deviation shaded in grey.

We collected large images containing multiple tissue cores, then accumulated single-point spectra as linescans across each individual core. Of the 8532 spectra that were collected, some were not representative of the tissue samples, as certain areas of the diagonal cross-sections contained blank ${\mathrm{CaF}}_{2}$, mainly at the sample’s edge (Figure 2). The spectra of the blank substrate were removed from the dataset. As shown in Figure 3, the raw spectra collected from the tissue samples were highly variable. The signal variation towards 1000 cm^−1^ can be related to the optical diffraction limit when using slits comparable to the wavelength, plus a dispersive effect due to the ${\mathrm{CaF}}_{2}$ substrate. It is well established that the transmission IR microscopy of tissue samples can suffer from significant baseline distortions from the basic offset, due to local optical density, predominantly resonant Mie scattering [57]. To combat this, an iterative EMSC approach was employed, as described elsewhere [48]. The resulting spectra were subjected to a PCA-quality test, described in Figure 4 (initial PCA plot can be found in Figure S1). All datapoints positioned outside of the centroid ellipse relate to spectra that had a significant scatter (gathered from rough sections of the TMA cores, e.g., the edges of the samples, or where no sample was present) and were removed from subsequent analysis. Additional quality testing was based on the intensity of the Amide I band, with only the spectra falling within an acceptance window of 0.01–2 being retained. The resulting spectra are outlined in Figure 5, where the baseline variation and scattering effects have been significantly reduced.

Following the data management described, 4822 spectra were retained for further spectral pre-processing and classification. In order to determine the optimal pre-processing parameters for the *IDH1*-mutated versus *IDH1*-wildtype dataset, a grid search was carried out using the PRFFECT toolbox, where the values for normalisation, binning, smoothing, order of derivative, and spectral cut were altered, as outlined in Table 2. A spectral cut of 1800–1200 cm^−1^ was included as there appeared to be a drop in signal below <1200 cm^−1^ for many samples, which was thought to be a result of the loss of IR light transmittance through the ${\mathrm{CaF}}_{2}$ slides (Figure S2). Thus, the fingerprint region with the removal of wavenumbers <1200 cm^−1^ was included in the grid search, along with the typical biological fingerprint region (1800–1000 cm^−1^) and the full spectral region. In total, 576 combinations of pre-processing parameters were tested.

The processed datasets were split 70:30 into training and testing sets. An LDA classifier was trained and predictions made on the testing set, resampled 11 times (i.e., repeated for 11 different train–test splits), and the classification probability threshold was chosen to optimise Cohen’s Kappa (κ). Briefly, the values of κ range from below zero to one, and measure the level of agreement between the classifier and the pathology, with higher values representing better agreement, thus signifying a more reliable diagnostic model [58]. The models predicted *IDH1* status on a ‘by sample’ basis, where the majority vote for each tissue core was reported as either *IDH1*-mutated or *IDH1*-wildtype. Here, the sensitivity is the ability to detect the positive class (the *IDH1* mutation), and specificity refers to *IDH1*-wildtype predictions. The results from all 11 iterations were averaged and compiled for comparison (Table S3).

**Figure 6:**
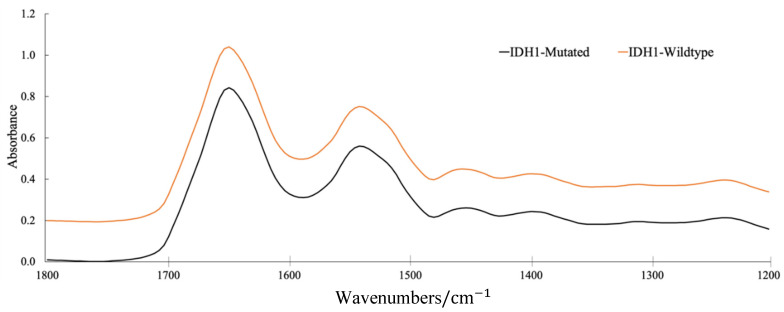
Mean pre-processed spectra for the synchrotron-based *IDH1* dataset, cut between 1800 and 1200 cm^−1^. Spectra offset for clarity; mutated (black) and wildtype (orange).

**Figure 7:**
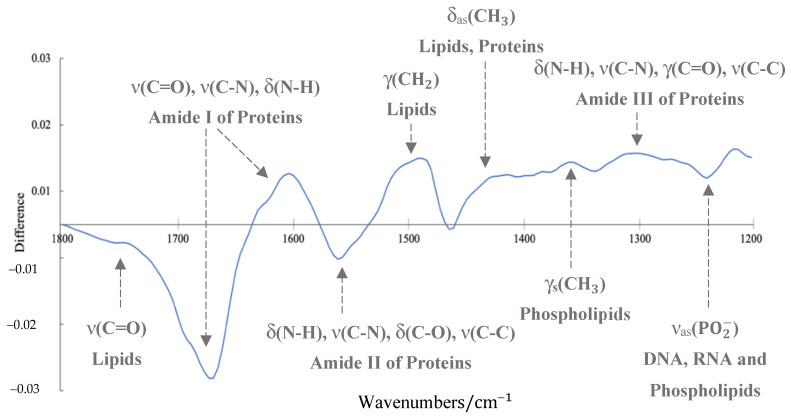
Difference spectrum of mean *IDH1*-mutated and *IDH1*-wildtype absorbance spectra for the synchrotron-based *IDH1* dataset, with tentative biological assignments and associated vibrational modes: ν = stretching; δ = bending; γ = wagging, twisting and rocking; as = asymmetric; s = symmetric.

The best performing model from the grid search reported a k value of 0.65, which demonstrates a substantial level of agreement [59]. This model provided a sensitivity and specificity of 87.8% and 86.2%, respectively, and the employed pre-processing was a min–max normalisation (i.e., scaling spectrum to between 0 and 1), followed by a binning factor of 4, a Savitzky–Golay (SG) filter with a filter length 7 and filter order 4, and a spectral cut to between 1800 and 1200 cm^−1^. The resulting mean spectra for both the *IDH1*-mutated and *IDH1*-wildtype patient groups are outlined in Figure 6. By plotting the difference between these mean spectra, it becomes evident that there are dissimilarities between the two *IDH1* groups (Figure 7).

A reliable classification result was anticipated since 11 resamples were employed in the grid search with different randomly selected training and test sets each time, and since the model reported a substantial level of agreement (k = 0.65). To ensure these findings were consistent, the top 10 pre-processing parameters from the grid search were further examined. Additionally, sampling techniques were utilised to combat the class imbalance between mutated and wildtype samples, ensuring no bias was present within the models. Each of the retained pre-processing combinations were classified by LDA with 51 resamples, and four sampling methods were tested in each instance: no additional sampling, up- and down-sampling, and synthetic minority over-sampling technique (SMOTE), based on our previous work [51]. The optimal pre-processing parameters from the initial grid search were also found to be the best in this case, when combined with additional up-sampling (Table S4). The diagnostic ability decreased slightly, with a reported 82.4% sensitivity and 83.4% specificity (Table 3). The standard deviation is much higher for the sensitivity than the specificity, as is further defined by the confusion matrices in Figure S3.

**Figure 8:**
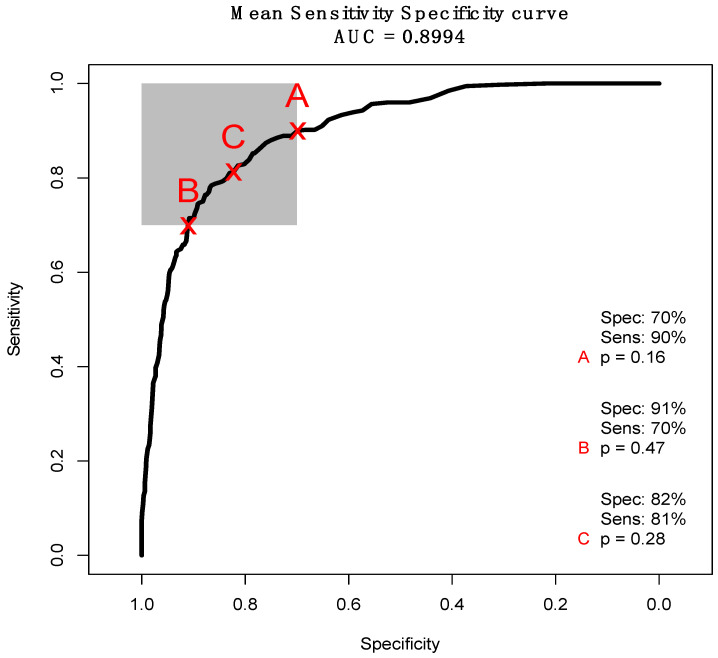
Mean receiver operator characteristic (ROC) curve displaying the trade-off between sensitivity and specificity for 51 resampled linear discriminant analysis (LDA) classifiers. The grey square is a target region of at least 70% for both sensitivity and specificity. The ‘x’ labels are the points on the curve that maximise sensitivity (A), specificity (B) and balance the two (C) whilst remaining in the target area, and ‘p’ represents the probability thresholds at those points on the curve.

Receiver operating characteristic (ROC) curves can also demonstrate a model’s diagnostic ability. Figure 8 describes a mean curve obtained from a resampled LDA ‘by sample’ classifier between *IDH1*-mutated and *IDH1*-wildtype. The curve is symmetrical across sensitivity and specificity, and reports an area under the curve (AUC) value of 0.8994, which is typically considered an excellent degree of discrimination between two classes [60]. By altering the probability threshold, denoted ‘p’ in Figure 8, we can maximise the sensitivity (A) or specificity (B), or obtain the greatest balance between the two (C). Point A represents the highest sensitivity (90%) whilst remaining in the 70% target region, whereas B denotes the maximum specificity (91%). The most balanced point on the curve used a probability threshold of 0.28 for every resample, and reported a sensitivity and specificity of 82% and 81%, respectively. This corroborates the high diagnostic ability of the model, and signifies some real promise for the determination of *IDH1* status through SR-FTIR microspectroscopy.

### 3.2. ATR-FTIR Results

Brain cancer patients, with either astrocytoma, oligodendroglioma or GBM, were separated based upon their *IDH1* status using ATR-FTIR serum spectroscopy. Of the 72 patients included, there were 36 with the *IDH1* mutation and 36 *IDH1*-wildtype. The data were classified through RF, PLS-DA and SVM with 100 resamples for each, and the findings are reported in Table 4 on a ‘by patient’ basis. For the whole serum dataset, the sensitivities were much higher than the specificities in each case. For example, the SVM model reported a promising sensitivity of 75.9%, but had an extremely low specificity of 28%.

**Figure 9:**
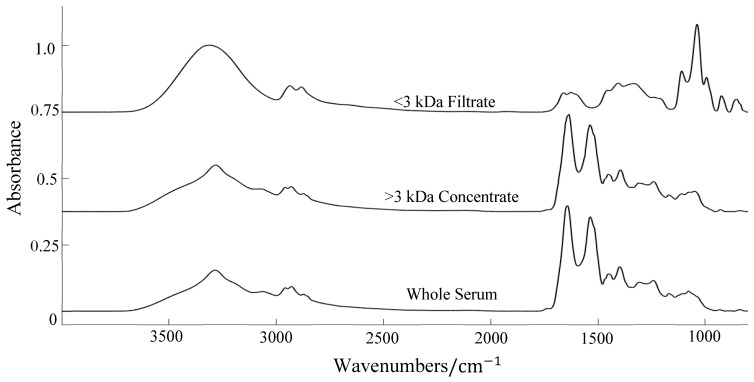
Examples of whole serum (bottom), the high molecular weight concentrate (middle) and the low molecular weight filtrate (top) spectra. Raw spectra offset for clarity.

Figure 9 provides an example of the IR spectra for whole serum, the >3 kDa ‘HMW’ fraction and the <3 kDa ‘LMW’ fraction. The concentrate appears almost identical to the whole serum spectrum; notably, they have a very similar absorbance to the more abundant proteins that exist within the Amide region, such as albumin and immunoglobulins. With these large proteins and other HMW constituents removed, the filtrate spectrum looks remarkably different, with only a few distinct peaks in the fingerprint region (<3 kDa, top spectrum). Three spectral regions were chosen for examination: 4000–800 cm^−1^ and 1800–800 cm^−1^, to encompass the two distinct peaks around 950 cm^−1^ and 850 cm^−1^, as well as the typical biological fingerprint region (1800–1000 cm^−1^). The classification results are reported in Table 5.

**Figure 10:**
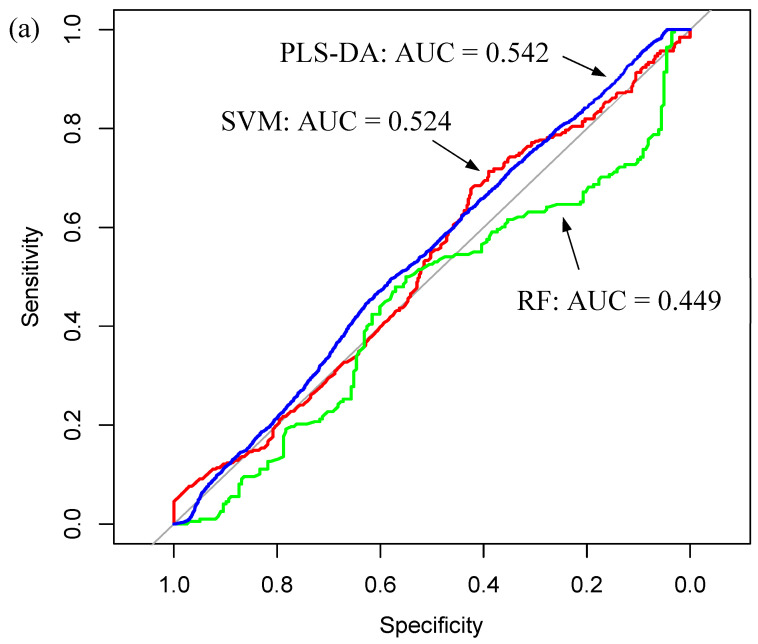

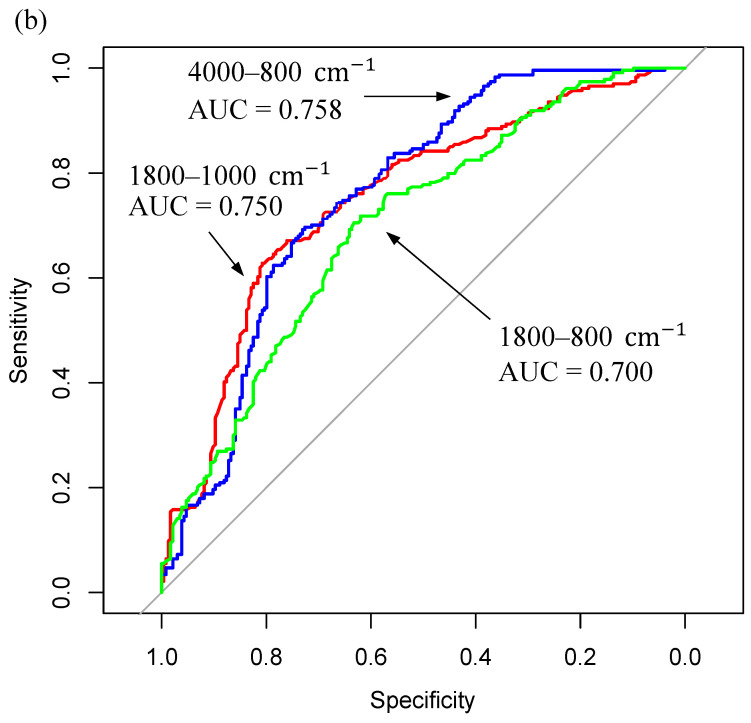
Single model receiver operator characteristic (ROC) graphs for the (**a**) whole serum dataset displaying the PLS-DA (blue), SVM (red) and RF (green) classifiers; and (**b**) the best performing model for each of the tested filtrate fractions: the full spectrum (4000–800 cm^−1^, blue), the fingerprint region (1800–1000 cm^−1^, red) and the extended fingerprint region (1800–800 cm^−1^, green).

In each case, the filtrate models were superior to the whole serum models in successfully detecting the *IDH1*-wildtype patients, reporting specificity values above 60%. The improvement in diagnostic ability due to the filtration step is emphasised in Figure 10, which displays single model ROC curves for the three whole serum classifiers (Figure 10a) and the best models for each of the three filtrate datasets (Figure 10b). As expected from the poor classification results, the ROC curves for the whole serum models fall on the diagonal line, meaning the predictions that are being made are no better than random guesses, and the reported AUC values of ~0.5 suggest the test has essentially no diagnostic accuracy [61]. However, the inclusion of centrifugal filtration enhanced the ability to successfully discriminate the two *IDH1* classes. The corresponding ROC curves in Figure 10b report AUC values >0.7, which is often deemed an acceptable level of discrimination [60].

**Figure 11:**
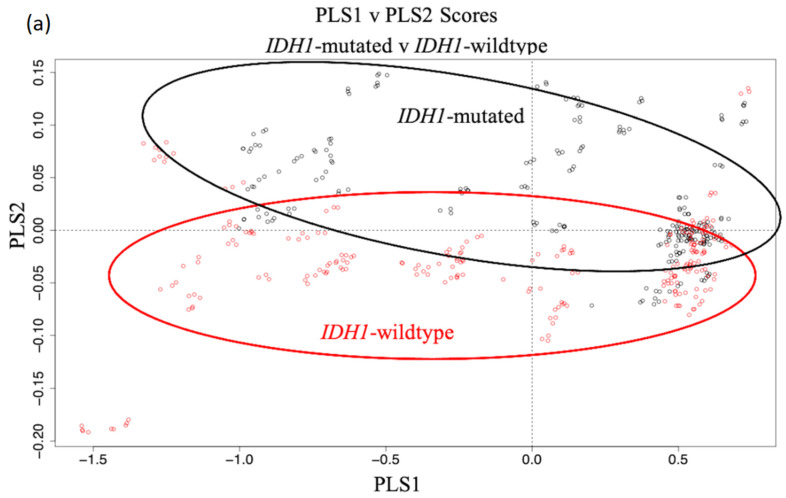

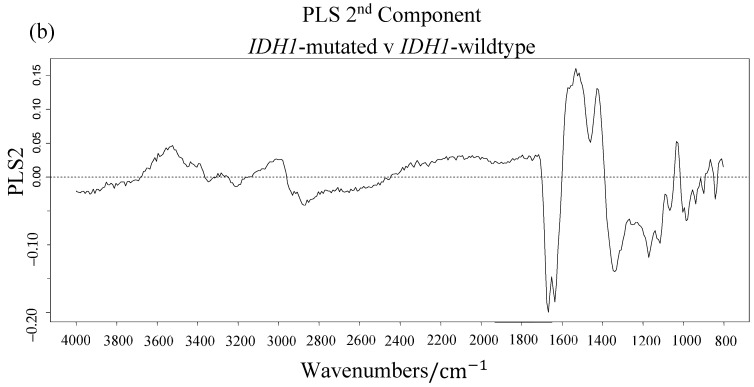
(**a**) the PLS scores plot between PLS1 and PLS2 for the *IDH1*-mutated (black) and *IDH1*-wildtype (red) data from the <3 kDa serum filtrate (4000–800 cm^−1^) dataset, and (**b**) the loadings for the 2^nd^ PLS component.

The <3 kDa filtered serum ‘full spectra’ dataset (4000–800 cm^−1^) delivered the greatest balanced accuracy of 69.1% when classified by the PLS-DA model. The PLS scores plot in Figure 11a describes the general variation within the dataset. The major variance is generally described by the first PLS component (PLS1). The PLS1 loadings suggest large differences of ~3400 cm^−1^ and ~1650 cm^−1^ (Figure S4), although there is no apparent class separation across PLS1 in the scores plot. Despite some overlap, it is evident that the second PLS component (PLS2) separates the two classes better than PLS1. The PLS2 loadings also highlight significant spectral differences around ~1650 cm^−1^ (Figure 11b). Interestingly, this is the typical location of the large Amide I band in a normal serum spectrum, accounting for the bond vibrations within an abundance of protein molecules. Even with the HMW proteins filtered out of the samples, such as albumin and immunoglobulins, it still appears to be a region of importance when examining molecules of very low molecular weights (<3 kDa), suggesting the smaller protein molecules still have diagnostic potential. Considerable contributions from lipids (~1450 cm^−1^), nucleic material (~1100 cm^−1^) and C-O-C stretching vibrations associated with carbohydrates and glycogen were apparent in the PLS2 loadings, as well as other proteinaceous vibrations (~1550 cm^−1^ and ~1300 cm^−1^).

The RF model for the 1800–800 cm^−1^ dataset also reported promising results, with a sensitivity and specificity of 70.6% and 66.4%, respectively. The Gini impurity metric was examined to identify the most important features within each dataset (Figure S5) [62]. Table 6 gives an overview of the top 15 identified wavenumbers in order of importance, with their corresponding wavenumber assignments and vibrational modes [27]. The top wavenumbers mostly account for the stretching vibrations of C-O, C-C and C-OH bonds, which are often associated with carbohydrates, glycogen and nucleic acids. Additionally, the symmetric ${\mathrm{PO}}_{2}^{-}$ stretching vibrations from DNA and ${\mathrm{CH}}_{2}$ twisting and bending vibrations associated with lipids were deemed significant in the RF classification. Likewise, wavenumbers in the Amide region were considered important here, accounting for C = O/C-N stretching and N-H bending vibrations in the amide bonds within protein molecules, similar to the PLS loadings described in Figure 11.

## 4. Discussion

In the synchrotron data analysis, a spectral cut of 1800–1200 cm^−1^ was included in the initial grid search as there appeared to be a drop in signal below <1200 cm^−1^. This cut-off effect for ${\mathrm{CaF}}_{2}$ in the low wavenumber region is common in scanning microscopy and can be caused by the change in refractive index (RI), e.g., the RI of ${\mathrm{CaF}}_{2}$ decreases from ~1.4 at 5 μm to ~1.3 at 10 μm. Additionally, when using a synchrotron source in the scanning microscopy mode for high spatial resolution, the diffraction limit is achieved when the microscope’s aperture defines a spot size scaled with the longest wavelength of the spectral region of interest [63]. Here, we used 10 μm slits, and therefore the diffraction limit could be affecting the signal towards 1000 cm^−1^ (full width half maximum (fwhm) ~l/2*NA = l = 10 μm, i.e., same size as the slit size used for scanning microspectroscopy) [64]. This did not appear to be a significant problem as the optimal pre-processing method involved the removal of this spectral region. 

In Figure 7, arguably the largest difference between the *IDH1*-mutated and *IDH1*-wildtype groups arises within the Amide I band, associated with the stretching of double-bonded carbonyl groups (C = O) and C-N bonds, as well as N-H bending vibrations in proteinaceous biomolecules [27]. The lower-wavenumber side of the Amide I band (1620–1600 cm^−1^) was more intense in the *IDH1*-mutated spectra (positive regions in the difference spectrum), whereas the band intensities between 1700 and 1650 cm^−1^ were lower compared to the *IDH1*-wildtype tissue spectra. This is consistent with a study wherein *IDH1*-mutated cell lines exhibited an elevated absorbance at 1610 cm^−1^, but a lower intensity around 1690 cm^−1^ [40]. These findings are not directly comparable to the results presented here, as cell lines may not adequately represent primary cells in clinical specimens [65]. The observed differences are likely to result from alterations in overlapping bands existing within the broad Amide I envelope, accounting for various protein secondary structures that can only be revealed with deconvolution techniques [66]. It is thought that the large negative peak in the difference spectrum at ~1660 cm^−1^ may represent a deviation in the levels of α-helical structures, and the smaller positive peak ~1615 cm^−1^ may be tentatively assigned to β-sheet components [67]. The band intensities at approximately ~1750 cm^−1^ and ~1560 cm^−1^ were lower in the mean *IDH1*-mutated spectrum, while those at ~1495 cm^−1^ and between 1450 and 1200 cm^−1^ displayed a higher absorbance than the *IDH1*-wildtype spectra (Figure 7).

Several spectral differences are similar to previous findings, namely, the variances in Amide III of proteins (mainly N-H in plane bending and C-N stretch, ~1300 cm^−1^), nucleic material such as DNA and RNA (${\mathrm{PO}}_{2}^{-}$ asymmetric stretch, ~1230 cm^−1^), and lipidic contributions (C = O stretch, ~1750 cm^−1^; CH_3_ bending, ~1450 cm^−1^) [40]. The disparities in the IR spectra could potentially be attributed to the increase in 2HG in the *IDH1*-mutated glioma tissue, which is known to be elevated in tumour cells with the *IDH1* mutation [8]. With reference to an IR spectrum of pure 2HG [40], the bands around 1589, 1450, 1416, 1344, 1311, 1267, 1236 and 1203 cm^−1^ could explain some of the differences observed between *IDH1*-mutated and *IDH1*-wildtype tissue in this study, as the band intensities at these wavenumbers are all elevated in *IDH1*-mutated patients, as described by the difference spectrum (Figure 7). That being said, it may only indicate a global change in biomolecular content, reflected by the systemic response of the genetic mutation within the glial tumour cells.

The disparities in the classification results between the initial grid search and the LDA model resampled 51 times highlight the importance of utilising a reasonable number of iterations, in order to minimise the variance whilst maintaining a respectable analysis time. However, the sensitivity and specificity remained well-balanced and above 80%, which are highly promising results. As shown in Table 3, the standard deviation is much higher for the sensitivity than the specificity, which is not entirely surprising because of the lower number of *IDH1*-mutated samples within the dataset. A 70:30 split between training and testing data meant that there were only seven randomly selected *IDH1*-mutated samples in each of the 51 resampled test sets. Therefore, when a known mutated tissue core is misdiagnosed as *IDH1*-wildtype, it has a substantial effect on the sensitivity. As described in the confusion matrices in Figure S3, there is a drop of ~15% in sensitivity when an *IDH1*-mutated sample is predicted wrongly. Conversely, there is only a ~4% difference in specificity with a misdiagnosed *IDH1*-wildtype, as there were 26 *IDH1*-wildtype samples in every test set. Thus, the addition of more glioma samples with the *IDH1* mutation would be beneficial for this analysis, in order to minimise the associated error. Nevertheless, these values demonstrate significant potential, and a mean balanced accuracy of 82.9% indicates synchrotron-based transmission FTIR is capable of identifying mutated and wildtype *IDH1* tumours.

Regarding the ATR-FTIR results, the whole serum classifiers seemed to be more effective at predicting the *IDH1*-mutated serum samples from the test sets, as the sensitivities were much higher than the specificities in each case. It is not clear why this may be, as there were an equal number of samples in each class, and therefore there should be no bias present in the models. That being said, the results did not appear to be reliable, and given the poor balanced accuracies (~50%), it could be assumed that the correct predictions were ultimately made by chance. Likewise, the low AUC values from the ROC curves (Figure 10) suggest they had no diagnostic ability. The LMW fraction of the serum is believed to contain disease-specific information, making the spectroscopic signature of this fraction useful for diagnostics [68]. Thus, after the poor classification performance for the whole serum data, it was thought that discrete molecular differences could potentially be emphasised through the use of centrifugal filtration. The balanced accuracies were enhanced to between 60 and 70% for all tested filtrate models. The centrifugal filtration step produced a significant improvement in the model’s performance, by delivering more balanced sensitivities and specificities. Similar to the tissue-based results, these findings are based on a relatively small cohort with only 36 patients in each class, thus misdiagnosed patients have a profound effect on the sensitivity or specificity values. Additional analysis with a larger patient cohort would be beneficial in identifying the true potential of the technique for this particular clinical application.

## 5. Limitations

Despite the promising results reported in this preliminary study, it is important to highlight some of the limitations. Since we have utilised the UK’s synchrotron facility here, the current methodology is not directly translatable to the hospital setting. Synchrotron instruments are admirable for high spatial resolution; thus, it was chosen in this project to attain the greatest level of biological and diagnostic detail from the glioma samples. Synchrotron measurements can be subject to lengthy analysis times, but standard bench-top FTIR spectrometers can acquire similar data quality with more efficient analysis. The ability to discriminate the *IDH1* mutation in glioma TMA sections with 80% accuracy would likely be clinically acceptable, although future studies should also consider probing fresh tissue biopsies rather than FFPE tissue microarrays, which would be better suited to the determination of a patient’s *IDH1* status mid-surgery. 

The whole serum results reported an accuracy of ~50%, which would not be deemed acceptable in the clinic. Blood serum comprises thousands of different proteins, ranging from the more abundant HMW serum albumin (50 g/L) to the LMW proteins like troponin (1 ng/L) [69]. Due to the wealth of various biomolecules that exist in a normal serum sample, it was expected to be a significant challenge to identify the subtle alterations in blood composition that may have been associated with the *IDH1* mutation. The filtration step did improve the classification performance, increasing the accuracy up to almost 70%. Although, it has been suggested elsewhere that the large absorbance band observed in the filtered serum spectrum (Figure 9, ~1030 cm^−1^) is due to glycerine interference, introduced into the sample from the centrifugal filters [70]. This could potentially be obscuring crucial information that may help improve the test performance. Future research could implement a washing step prior to centrifugation. There are also many filter sizes to choose from, hence filtration with a different cut-off point may also further improve classification performance. Many cytokines and chemokines exist at molecular weights greater than 3kDa, which may be indicative of disease.

As already stated, both the tissue and serum analysis could benefit through the addition of more patients, preferably from prospective trials, which would likely reduce the standard error within the classification models. It is vital that more efficient methods are developed for this application before clinical translation can be realised.

## 6. Conclusions

FTIR spectroscopy during surgical tissue biopsy as a label-free test for the molecular genetic classification of gliomas could impact on surgical decision-making, in particular about the extent of resection. The initial synchrotron-based microanalysis reported 87.8% sensitivity and 86.2% specificity. Further examination utilising a higher number of resamples slightly reduced the diagnostic outcome, with 51 LDA iterations reporting a balanced accuracy of 82.9%. ROC analysis produced a mean curve with an AUC of 0.8994, which also suggests a good degree of diagnostic separability. This demonstrates significant potential for detecting the molecular alterations initiated by genetic mutations in the *IDH1* enzyme.

Identification of the molecular status from blood serum prior to biopsy could further direct some patients to alternative treatment strategies. Initially, the whole serum classifiers performed poorly, delivering balanced accuracies of ~50%. Yet with the introduction of centrifugal filtration, the classification performance improved significantly, enhancing the sensitivities and specificities to around 70%. These strategies can now be validated and optimised in prospective clinical studies, and can be extended to identify other important molecular alterations, such as *ATRX* loss, *1p/19q* co-deletion and/or *MGMT* hypermethylation, with which brain cancer type can be stratified pre-operatively.

## Figures and Tables

**Table 1:** Common genetic and chromosomal aberrations associated with the major glioma subtypes [6]. Abbreviations defined below the table.

Glioma Entity	WHO Grade	*IDH1* Mutation	Additional Associated Alterations
Pilocytic astrocytoma	I	Extremely rare	*BRAF*, *KRAS*, *NF1*, *FGFR1*
Diffuse astrocytoma	II	Common	*IDH2*, *TP53*, *ATRX*, *LOH 17p*
Anaplastic astrocytoma	III	Common	*IDH2*, *TP53*, *ATRX*, *LOH 17p*
Oligodendroglioma	II	Majority of cases	*IDH2*, *1p/19q co-deletion*
Anaplastic oligodendroglioma	III	Majority of cases	*IDH2*, *1p/19q* co-deletion
Glioblastoma (primary)	IV	Rare	*TERT*, *PTEN*, *TP53*, *MGMT* hypermethylation, *EGFR*, *7+/10−*
Glioblastoma (secondary)	IV	Extremely Common	*IDH2*, *TP53*, *ATRX*, *LOH 17p*

*NF1*, neurofibromatosis type 1; *FGFR1*, fibroblast growth receptor 1; *IDH2*, isocitrate dehydrogenase 2; *TP53*, tumour suppressor protein 53; *ATRX*, alpha thalassemia/mental retardation syndrome X-linked mutation; *LOH 17p*, loss of heterozygosity on chromosome 17; *TERT*, telomerase reverse transcriptase; *PTEN*, phosphatase and tensin homolog; *MGMT*, O(6)-methlyguanine-DNA-methyltransferase; *EGFR*, epidermal growth factor receptor, 7+/10−, gain of chromosome 7 and loss of chromosome 10. Italics: Genes.

**Table 2:** Pre-processing parameters examined in machine learning grid search.

Parameter	Variations			
Normalisation (n)	None (0)	Min-max (1)	Vector (2)	Amide I (3)
Derivative (l)	None (0)	First (1)	Second (2)	-
Binning (b)	1	2	4	8
Smoothing with Savitzky–Golay filter (s)	None (0)	2	3	4
Spectral cut (p)	None (0)	1800–1000 cm^−1^	1800–1200 cm^−1^	-

**Table 3:** Classification results from 51 resamples of the optimal LDA model with additional up-sampling, in terms of sensitivity, specificity and balanced accuracy.

Statistic	Mean	Standard Deviation
Sensitivity (%)	82.4	16.8
Specificity (%)	83.4	8.2
Balanced Accuracy (%)	82.9	9.6

**Table 4:** Classification results for the *IDH1*-mutated versus *IDH1*-wildtype whole serum dataset, after 100 resamples. The mean sensitivity, specificity and balanced accuracy are reported with their corresponding standard deviations (SD).

Sample Fraction	Model	Sensitivity (%)		Specificity (%)		Balanced Accuracy (%)	
		Mean	SD	Mean	SD	Mean	SD
Whole Serum	RF	50.3	15.2	45.4	15.1	47.9	8.6
	PLS-DA	69.3	13.8	35.3	14.7	52.3	7.4
	SVM	75.9	17.5	28.0	14.6	51.9	7.7

**Table 5:** Classification results for the *IDH1*-mutated versus *IDH1*-wildtype serum datasets after 100 resamples. The mean sensitivity, specificity and balanced accuracy are reported with their corresponding standard deviations (SD). The best performing model for each sample fraction is highlighted in bold.

Sample Fraction	Model	Sensitivity (%)		Specificity (%)		Balanced Accuracy (%)	
		Mean	SD	Mean	SD	Mean	SD
**<3kDa Filtered Serum (4000–800 cm^−1^)**	RF	68.4	16.2	67.5	15.9	68.0	11.1
	**PLS-DA**	**75.5**	12.3	**62.6**	15.5	**69.1**	9.0
	SVM	68.4	16.5	64.2	16.0	66.4	10.2
<3kDa Filtered Serum (1800–800 cm^−1^)	**RF**	**70.6**	17.8	**66.4**	14.5	**68.5**	11.2
	PLS-DA	65.0	14.6	64.6	16.5	64.8	8.7
	SVM	63.2	16.3	63.8	16.9	63.5	9.6
<3kDa Filtered Serum (1800–1000 cm^−1^)	**RF**	**66.6**	15.4	**68.1**	14.1	**67.4**	9.9
	PLS-DA	65.9	14.6	56.2	15.5	61.1	9.1
	SVM	68.1	15.6	56.8	15.6	62.5	10.1

Bold: the best performing models.

**Table 6:** The top 15 wavenumbers from the <3 kDa serum filtrate (1800–800 cm^−1^) random forest classification between *IDH1*-mutated and *IDH1*-wildtype with associated vibrational modes [27]. The column “ΣGini” is a summation of the mean decrease in Gini for each wavenumber, over all nodes in all trees in the random forest ensemble, which suggests the regions of highest importance.

$\mathrm{Wavenumbers}\;({\mathrm{cm}}^{-1})$	∑ Gini	Vibrational Modes
1124.5	12.31	C-O stretch
1172.5	11.22	C-O, C-OH stretch
1164.5	9.07	C-C, C-O and C-OH stretch
1180.5	6.43	${\mathrm{CH}}_{2}$ twisting
1116.5	5.39	RNA; C-OH stretch
1028.5	5.01	Carbohydrate; C-O stretch
1188.5	4.46	DNA; Symmetric ${\mathrm{PO}}_{2}^{-}$ stretch
1740.5	4.19	Lipids; C = O stretch
1020.5	3.60	Glycogen; C-O stretch
1132.5	3.49	C-O and C-C stretch
1588.5	2.77	Amide I; C = O and C-N stretch, N-H bending
1548.5	2.73	Amide II; N-H bending, C-N stretching
1444.5	2.57	Lipids; ${\mathrm{CH}}_{2}$ bending
1468.5	2.52	Lipids/Proteins; ${\mathrm{CH}}_{2}$ bending
1612.5	2.45	Amide I; C = O and C-N stretch, N-H bending

## Supplementary Materials

The following are available online at https://www.mdpi.com/2072-6694/12/12/3682/s1, Figure S1. PCA-based quality test: PCA scores plot of PC1 and PC2 before selection of central cluster. Figure S2. (a) Absorbance spectrum of blank ${\mathrm{CaF}}_{2}$ substrate and (b) raw sample spectra affected by change in absorbance baseline <1200 cm^−1^. Figure S3. Confusion matrices showing the predictions of two of the randomly selected test sets in from the linear discriminant analysis classification. Figure S4. The loadings plot for the 1^st^ PLS component highlighting differences between *IDH1*-mutated and *IDH1*-wildtype, for the <3 kDa serum filtrate (4000–800 cm^−1^) dataset. Figure S5. Random forest Gini importance plots for the <3 kDa filtrate datasets, showing the most important wavenumbers responsible for the *IDH1*-muated versus *IDH1*-wildtype classifications; (a) 4000–800 cm^−1^, (b) 1800–800 cm^−1^ and (c) 1800–1000 cm^−1^. Table S1. Samples included in the centrifugal filtration of serum study. Table S2. Samples included in the synchrotron-based tissue microarray study. Table S3. Top 50 LDA models from pre-processing grid search, based on Kappa score. Table S4. Top 10 LDA models from pre-processing grid search, with sensitivity and specificity results from 51 resamples. Optimal results in bold.
