# Supplementary material for: Interrogation of IDH1 Status in Gliomas by Fourier Transform Infrared Spectroscopy
Source: Cancers (Basel). 2020 Dec 8;12(12):3682. doi: 10.3390/cancers12123682 (PMC7762605; doi:10.3390/cancers12123682)
Supplement: Supplementary file 1 [file cancers-12-03682-s001.pdf]

cancers

Supplemental Materials

Interrogation of *IDH1* status in gliomas by Fourier transform infrared spectroscopy

James M. Cameron, Justin J.A. Conn, Christopher Rinaldi, Alexandra Sala, Paul M. Brennan, Michael D. Jenkinson, Helen Caldwell, Gianfelice Cinque, Khaja Syed, Holly J. Butler, Mark G. Hegarty, David S. Palmer, Matthew J. Baker

Table S1. Samples included in the centrifugal filtration of serum study.

	IDH1-mutated	IDH1-wildtype
**Total**	36	36
**Sex (M/F)**	21 / 15	23 / 13
**Glioblastoma**	0	12
**Astrocytoma**	24	24
**Oligodendroglioma**	12	0

Table S2. Samples included in the synchrotron-based tissue microarray study.

	IDH1-mutated	IDH1-wildtype
**Total**	21	78
**Sex (M/F)**	9 / 12	48 / 30
**Glioblastoma**	10	67
**Astrocytoma**	4	4
**Pilocytic Astrocytoma**	0	2
**Oligodendroglioma**	7	5

Table S3. Top 50 LDA models from pre-processing grid search, based on Kappa score.

model	Accuracy	Kappa	Sensitivity	Specificity	F1	Balanced Accuracy
n1_l0-0_b4_s1-4_p1-1200to1800	0.8652038	0.6468702	0.8787879	0.8616601	0.732303	0.870224
n2_l0-0_b2_s1-3_p1-1200to1800	0.8902821	0.6314261	0.6515152	0.9525692	0.69312	0.8020422
n0_l1-0_b4_s0-0_p1-1200to1800	0.8652038	0.6166079	0.7424242	0.8972332	0.7005675	0.8198287
n3_l2-0_b8_s1-2_p1-1000to1800	0.8777429	0.5954007	0.6363636	0.9407115	0.6674396	0.7885375
n3_l1-0_b4_s1-2_p1-1000to1800	0.8714734	0.5936348	0.6515152	0.9288538	0.6720128	0.7901845
n3_l0-0_b4_s1-2_p1-1200to1800	0.8714734	0.591284	0.6363636	0.9328063	0.6641601	0.784585
n2_l2-0_b2_s0-0_p1-1200to1800	0.8652038	0.5873832	0.6515152	0.9209486	0.6704387	0.7862319
n2_l0-0_b4_s1-3_p1-1000to1800	0.8432602	0.5841362	0.8030303	0.8537549	0.6837897	0.8283926
n1_l0-0_b4_s1-2_p1-1000to1800	0.862069	0.5782494	0.6666667	0.9130435	0.6597425	0.7898551
n2_l0-0_b4_s1-4_p1-1200to1800	0.8526646	0.5766405	0.7424242	0.8814229	0.6684959	0.8119236

n3_l2-0_b4_s1-2_p1-1200to1800	0.8369906	0.5737398	0.8181818	0.8418972	0.6777389	0.8300395
n1_l0-0_b1_s0-0_p1-1200to1800	0.8557994	0.5722462	0.6969697	0.8972332	0.66271	0.7971014
n3_l2-0_b1_s1-4_p1-1200to1800	0.8652038	0.5707929	0.6060606	0.9328063	0.6527745	0.7694335
n1_l1-0_b4_s0-0_p1-1000to1800	0.8495298	0.5705938	0.7121212	0.8853755	0.6595468	0.7987484
n3_l1-0_b4_s1-2_p1-1200to1800	0.8714734	0.5653157	0.5757576	0.9486166	0.6402981	0.7621871
n2_l1-0_b4_s1-3_p1-1200to1800	0.8683386	0.5632158	0.6060606	0.9367589	0.6385675	0.7714097
n1_l0-0_b4_s1-3_p1-1000to1800	0.8777429	0.5604897	0.530303	0.9683794	0.6263545	0.7493412
n1_l2-0_b4_s0-0_p1-1200to1800	0.8401254	0.5600921	0.7272727	0.8695652	0.6606487	0.798419
n2_l1-0_b4_s0-0_p1-1000to1800	0.862069	0.5597247	0.6060606	0.9288538	0.6414767	0.7674572
n2_l2-0_b4_s1-2_p1-1200to1800	0.8714734	0.5571603	0.5757576	0.9486166	-	0.7621871
n1_l1-0_b4_s1-4_p1-1200to1800	0.8432602	0.5571262	0.7121212	0.8774704	0.655943	0.7947958
n0_l2-0_b8_s1-3_p1-1000to1800	0.8777429	0.5568751	0.5	0.9762846	0.6216713	0.7381423
n3_l2-0_b8_s1-4_p1-1000to1800	0.8526646	0.556261	0.6515152	0.9051383	0.647619	0.7783267
n0_l1-0_b4_s1-3_p1-1200to1800	0.8401254	0.5533281	0.6818182	0.8814229	0.6529921	0.7816206
n0_l0-0_b1_s1-4_p1-1200to1800	0.8714734	0.5528742	0.530303	0.9604743	0.624864	0.7453887
n3_l0-0_b4_s1-3_p1-1200to1800	0.8557994	0.5504227	0.5909091	0.9249012	0.637072	0.7579051
n0_l2-0_b2_s1-2_p1-1200to1800	0.8495298	0.5495883	0.6666667	0.8972332	0.6441195	0.7819499
n1_l0-0_b1_s1-4_p1-1200to1800	0.8557994	0.5464795	0.6212121	0.916996	0.633856	0.7691041
n1_l2-0_b4_s1-3_p1-1000to1800	0.830721	0.5452847	0.7727273	0.8458498	0.6522455	0.8092885
n1_l0-0_b4_s1-2_p1-1200to1800	0.8275862	0.5450624	0.7727273	0.8418972	0.6542278	0.8073123
n2_l0-0_b2_s1-4_p1-1200to1800	0.8338558	0.5411106	0.7272727	0.8616601	0.6446316	0.7944664
n3_l1-0_b4_s1-3_p1-1200to1800	0.815047	0.5410151	0.8030303	0.8181818	0.6568059	0.8106061
n1_l2-0_b2_s1-2_p1-1200to1800	0.8401254	0.5357673	0.6818182	0.8814229	0.6363213	0.7816206
n0_l1-0_b4_s1-3_p1-1000to1800	0.8526646	0.5351708	0.6212121	0.9130435	0.6204923	0.7671278
n0_l2-0_b4_s1-3_p1-1200to1800	0.8369906	0.5350227	0.6969697	0.8735178	0.6352135	0.7852437

n2_l0-0_b4_s1-3_p1-1200to1800	0.8746082	0.5348123	0.4848485	0.9762846	0.5971861	0.7305665
n0_l0-0_b2_s1-3_p1-1200to1800	0.8495298	0.5345926	0.6212121	0.9090909	0.6280417	0.7651515
n1_l2-0_b8_s0-0_p1-1000to1800	0.8589342	0.5339241	0.5757576	0.9328063	0.6160536	0.7542819
n3_l1-0_b4_s1-4_p1-1200to1800	0.846395	0.5314692	0.6363636	0.9011858	0.6227329	0.7687747
n1_l1-0_b4_s1-3_p1-1200to1800	0.8714734	0.5313889	0.4848485	0.972332	0.5975075	0.7285903
n1_l2-0_b8_s1-3_p1-1200to1800	0.8432602	0.5280296	0.6515152	0.8932806	0.6196915	0.7723979
n1_l1-0_b4_s1-2_p1-1200to1800	0.8087774	0.5278049	0.7878788	0.8142292	0.6458796	0.801054
n3_l1-0_b4_s0-0_p1-1200to1800	0.8557994	0.5273934	0.5606061	0.9328063	0.6129608	0.7467062
n0_l1-0_b4_s1-4_p1-1200to1800	0.830721	0.5270823	0.7121212	0.8616601	0.6339407	0.7868906
n0_l2-0_b4_s1-2_p1-1200to1800	0.846395	0.5264629	0.6212121	0.9051383	0.6214876	0.7631752
n1_l0-0_b4_s1-4_p1-1000to1800	0.7899687	0.525957	0.8787879	0.7667984	0.6536246	0.8227931
n0_l2-0_b1_s1-4_p1-1200to1800	0.8369906	0.5235235	0.6818182	0.8774704	0.6248756	0.7796443
n0_l1-0_b4_s1-2_p1-1200to1800	0.8056426	0.5232485	0.8333333	0.798419	0.6443412	0.8158762
n1_l1-0_b1_s1-2_p1-1200to1800	0.8432602	0.5200658	0.6363636	0.8972332	0.6173735	0.7667984
n0_l2-0_b8_s1-4_p1-1000to1800	0.8369906	0.5200327	0.6515152	0.8853755	0.6224957	0.7684453

Table S4. Top 10 LDA models from pre-processing grid search, with sensitivity and specificity results from 51 resamples. Optimal results in bold.

Model Name	**Pre-processing**					**Sampling**			
	**Norm**	**Deriv**	**Bin**	**Smooth**	**Cut**	**No**	Up	Down	Smote
	**-**					**Sensitivity / Specificity (%)**			
n0_l1-0_b4_s0-0_p1-1200to1800	0	1	4	0	1200-1800	67.3/86.5	69.9/84.6	82.3/76.6	82.3/77.6
n0_l1-0_b4_s1-2_p1-1200to1800	0	1	4	2	1200-1800	76.1/83.5	89.5/72.0	87.9/70.4	81.7/78.4
n0_l2-0_b2_s1-4_p1-1200to1800	0	2	2	4	1200-1800	78.4/73.0	80.4/70.4	78.4/70.0	78.4/70.0
n1_l0-0_b4_s1-4_p1-1200to1800	1	0	4	4	1200-1800	76.5/81.3	**82.4/83.4**	78.1/85.0	80.7/79.5
n1_l0-0_b4_s1-4_p1-1000to1800	1	0	4	4	1000-1800	89.2/62.0	79.7/77.3	83.0/72.5	76.5/80.3
n2_l0-0_b2_s1-3_p1-1200to1800	2	0	2	3	1200-1800	86.6/68.7	82.7/72.7	80.1/76.8	79.7/75.2
n2_l0-0_b4_s1-3_p1-1000to1800	2	0	4	3	1000-1800	81.7/67.0	74.5/77.2	75.2/78.4	79.7/75.7
n2_l0-0_b4_s1-4_p1-1200to1800	2	0	4	4	1200-1800	82.0/75.1	88.6/71.7	88.2/69.5	87.6/73.1
n3_l1-0_b4_s1-2_p1-1000to1800	3	1	4	2	1000-1800	85.0/65.5	71.2/83.5	88.6/63.5	83.7/73.6
n3_l2-0_b4_s1-2_p1-1200to1800	3	2	4	2	1200-1800	81.7/78.3	87.3/73.8	80.7/76.2	76.8/79.4

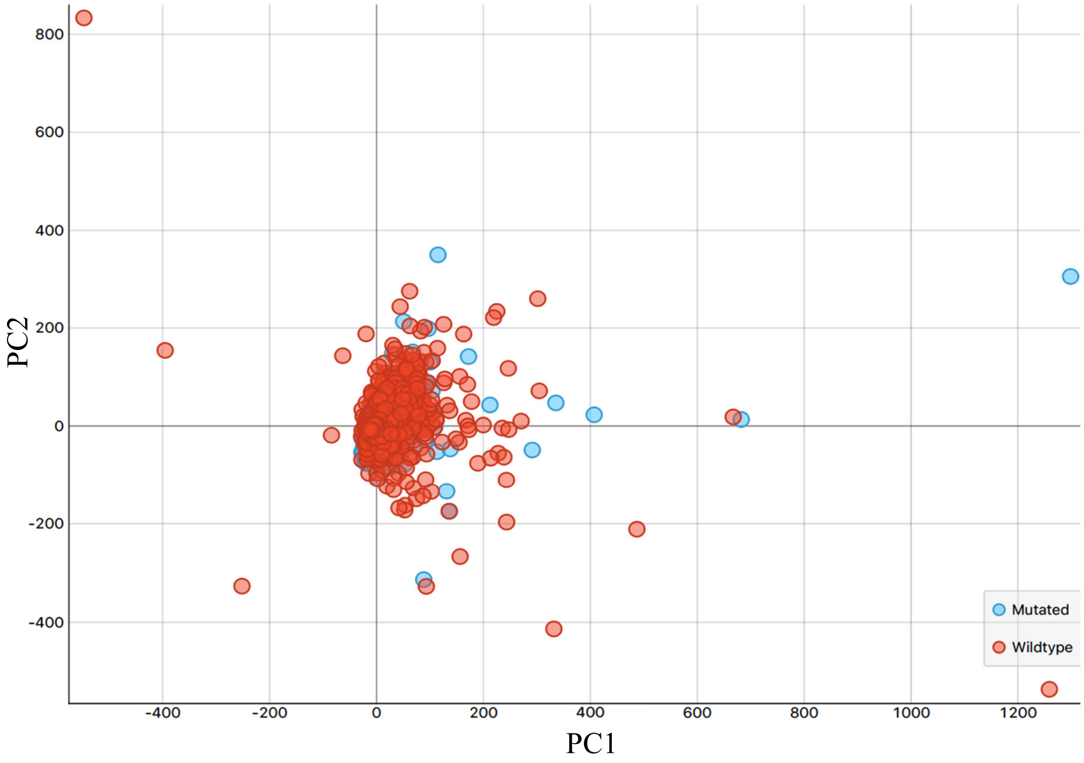

Figure S1. - PCA-based quality test: PCA scores plot of PC1 and PC2 before selection of central cluster.

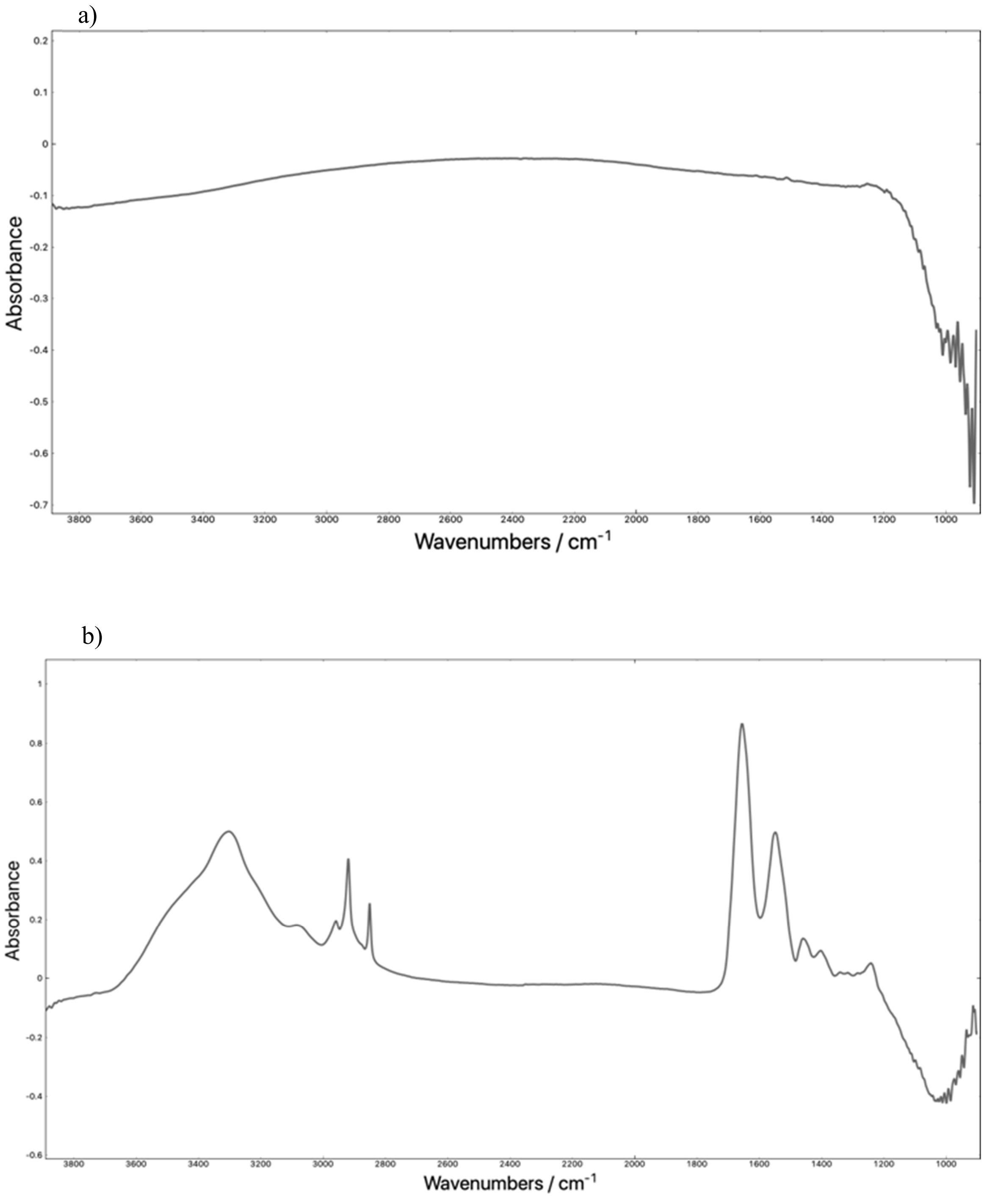

Figure S2. - a) Absorbance spectrum of blank CaF_2substrate and b) raw sample spectra affected by change in absorbance baseline <1200 cm^{-1}.

Prediction		Reference: Mutated	Reference: Wildtype
	Mutated	7	3
	Wildtype	0	23

Sensitivity = 100%   Specificity = 88.5%

Prediction		Reference: Mutated	Reference: Wildtype
	Mutated	6	2
	Wildtype	1	24

Sensitivity = 85.7%   Specificity = 92.3%

Figure S3. - Confusion matrices showing the predictions of two of the randomly selected test sets in from the linear discriminant analysis classification.

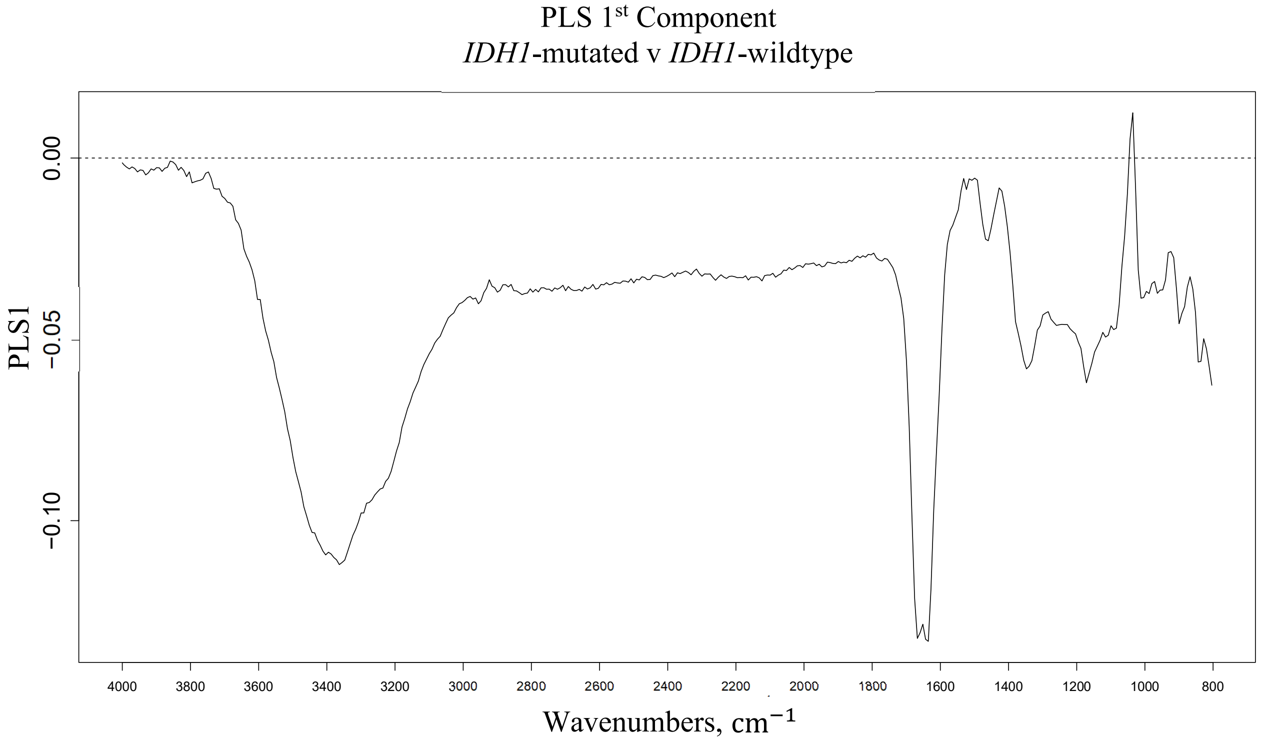

Figure S4. - The loadings plot for the 1st PLS component highlighting differences between *IDH1*-mutated and *IDH1*-wildtype, for the <3kDa serum filtrate (4000-800 cm^{-1}) dataset.

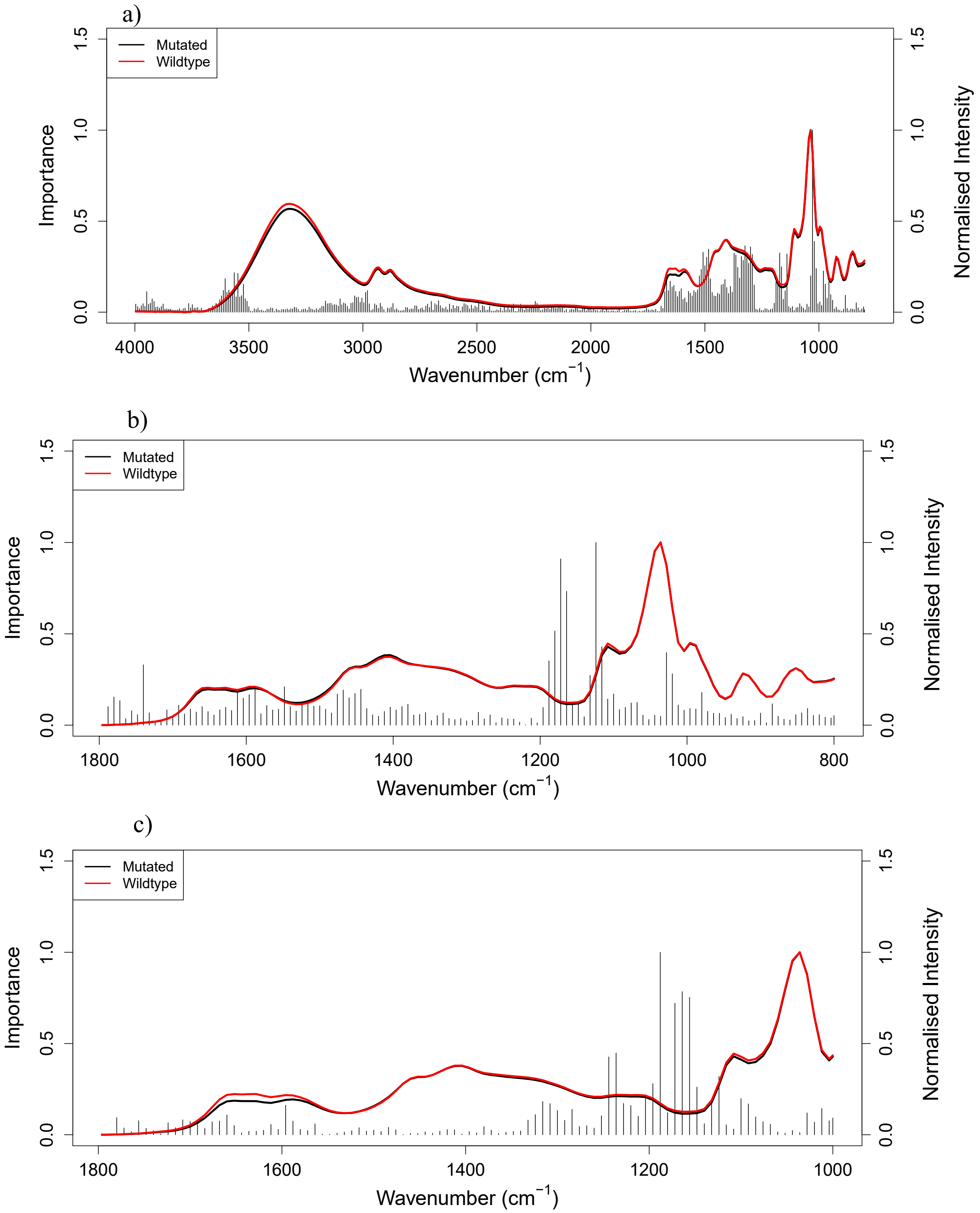

Figure S5. – Random forest Gini importance plots for the <3kDa filtrate datasets, showing the most important wavenumbers responsible for the *IDH1*-muated versus *IDH1*-wildtype classifications; a) 4000-800 cm^{-1}, b) 1800-800 cm^{-1} and c) 1800-1000 cm^{-1}.
